# Assessment of a novel electrochemically deposited smart bioactive trabecular coating (SBTC®): a randomized controlled clinical trial

**DOI:** 10.1186/s13005-024-00426-0

**Published:** 2024-04-16

**Authors:** Mark Adam Antal, Ramóna Kiscsatári, Gábor Braunitzer, József Piffkó, Endre Varga, Noam Eliaz

**Affiliations:** 1 Department of Operative and Esthetic Dentistry, Faculty of Dentistry, University of Szeged, 6720 Tisza Lajos Krt. 64-66, Szeged, Hungary; 2 Department of Oral and Maxillofacial Surgery, Faculty of Medicine, University of Szeged, Szeged, Hungary; 3 dicomLAB Dental, Ltd, Szeged, Hungary; 4 Department of Materials Science and Engineering, Tel-Aviv University, Tel Aviv, Israel

**Keywords:** Dental implant, Surface treatment, Osseointegration, RFA, Hydroxyapatite

## Abstract

**Objectives:**

A randomized controlled clinical trial of dental implants was conducted to compare the clinical properties of a novel electrochemically deposited calcium phosphate coating to those of a common marketed surface treatment.

**Material and methods:**

Forty implants of the same brand and type were placed in 20 fully edentulous participants requiring mandibular implantation. The two study groups were defined by the surface treatment of the implants. 20 implants in the control group were coated via a commercial electrochemical surface treatment that forms a mixture of brushite and hydroxyapatite, while the remaining 20 in the test group were coated with a novel electrochemical Smart Bioactive Trabecular Coating (SBTC®). A split-mouth design was employed, with each participants receiving one control implant in one mandibular side and a test implant in the other. To mitigate potential operator-handedness bias, control and test implants were randomly assigned to mandibular sides. All cases underwent digital planning, implant placement with a static surgical guide, and participants received locator-anchored full-arch dentures. The primary outcome was implant stability (measured using Osstell ISQ) assessed at insertion, loading, and then 3 months, 9 months, and 2 years post-insertion. The secondary outcome was bone level change (in millimeters) over the 2-year observation period. Oral health-related quality of life (OHRQL) was monitored using the OHIP-14 questionnaire. Complications and adverse events were recorded.

**Results:**

Successful osseointegration and implant stability were achieved in all cases, allowing loading. ISQ values steadily increased throughout the observation period. While no significant differences were observed between the SBTC® and control coatings, the test group exhibited a higher ISQ gain. Bone resorption was somewhat lower in the SBTC® but not significantly so. Patients' OHRQL significantly improved after denture delivery and remained stable throughout the follow-up. No complications or adverse events were observed.

**Conclusions:**

Based on the study results, we conclude that the new surface treatment is a safe alternative to the widely used control surface, demonstrating similar osseointegrative properties and time-dependent bone level changes. Further research may explore the broader implications of these findings.

**Trial registration:**

The study is registered on clinicaltrials.gov under the identifier ID: NCT06034171.

## Introduction

Tooth loss is a highly prevalent issue that can stem from factors such as decay, periodontal disease, or even trauma. Dental implants have become an established solution for replacing lost teeth in modern dentistry [1–5].

It is now generally accepted that the primary determinant of implant success is osseointegration [6]. Numerous studies have sought innovations aimed at reducing healing time and/or promoting osseointegration [7–9]. One of the most critical factors for successful osseointegration is the implant surface. There are several methods for modifying the surface of titanium implants, for example coating with calcium phosphate films or nanoparticles [10–12] or self-assembled monolayers (SAMs) [13–15].

Overall, hydrophilic surfaces (i.e. with high surface energy) appear to be more desirable than hydrophobic ones as they have shown more rapid cell activation and differentiation and positively affect adhesion and proliferation of osteoblasts [10, 16–20]. It has also been documented that a hydrophilic surface can lead to a higher level of bone-to-implant contact [16]. Numerous studies have confirmed that the surface roughness of implants influences osseointegration and biomechanical fixation [10, 21, 22]. Research suggests that both early stability and long-term success can be positively influenced by a rougher implant surface compared to a smooth one [10, 23, 24].

One approach to creating a rougher implant surface is through subtractive processes, such as sandblasting with or without acid-etching [25]. Another method is additive processes, like plasma spray (PS) of titanium or calcium phosphate (CaP) coatings [10, 26, 27]. In this category, hydroxyapatite (HAp) [10] has shown the best results, despite some findings suggesting that PS’ed HAp consistently showed signs of resorption [21, 25]. HAp has become a popular coating because its chemical composition and microscopic crystal structure are similar to those of natural bone [10, 28–30]. Several research groups have attempted various combinations of this, such as combining it with chitosan [12, 31], carbon-based materials [30, 32, 33] hyaluronic acid [34], and alginic acid [35]. Some studies have confirmed that CaP based surface treatments may possess osteoconductive properties [10, 36]. CaPs represent a family of materials consisting of various phases, including HAp, α- and ß-tricalcium phosphate, brushite, and octacalcium phosphate (OCP) [10]. Schiegnitz et al. demonstrated in an animal study that CaP-coated surfaces on supracrestal inserted implants exhibit vertical osteoconductive characteristics and significantly increase bone/implant contact at the implant shoulder [37].

These surface treatments have shown promising osteoconductive properties in numerous short-term animal experiments [6, 38, 39]. However, long-term clinical studies have raised concerns regarding degradation, delamination, osteolysis, and third-body wear as potential long-term effects [10, 40–42]. It is worth noting that in the PS process various non-stoichiometric, partially crystalline and amorphous phases are formed on the implant's surface, and residual stresses are introduced, ultimately leading to delamination of the coating and failure [10, 43, 44].

Bonit®, a commercially available electrochemical CaP coating, served as control in this study. According to its specification, this is a biphasic coating in which the more soluble outer phase (brushite, CaHPO_4_∙2H_2_O, Ca/P = 1.0) promotes short-term bone synthesis, whereas the inner phase (microcrystalline HAp) is resorbed more slowly and releases ions over a relatively long period. The Ca/P atomic ratio in this coating is 1.1 ± 0.1, the porosity level is 60%, the coating thickness is 20 ± 10 µm, and the adhesion strength is higher than 15 MPa. This value of the adhesion strength represents the minimum required by international standards [45]. One should also note the wide distribution of coating thickness (which is yet considerably thinner than the typical 50–200 μm thickness of PS’ed CaP coatings). It was reported that brushite can either convert in vivo into precipitated HAp (pHAp) or be degraded and replaced by bone [10]. When large amounts of brushite are converted into pHAp in vivo, a severe inflammatory response might be observed due to the large amounts of acid that are released during this reaction [46]. Reigstad et al. demonstrated in an animal study that after 6-week implantation, the bone/implant contact was higher for such coated implants compared to uncoated implants of the same shape made of Ti6Al4V [47].

The referenced studies and clinical practice both suggest that electrochemically deposited CaP surfaces provide good osseointegration with lower occurrence and delamination and cracking compared to PS’ed Hap [10, 43, 44]. The advantages of electrodeposited CaP (EDCaP) coatings include: (i) low process temperatures, which enable formation of highly crystalline deposits with low solubility in body fluids and low residual stresses; (ii) the ability to coat porous, geometrically complex, or non-line-of-sight surfaces; (iii) the possible improvement of the substrate/coating bond strength; (iv) the ability to control the thickness, composition and microstructure of the deposit; and (v) the ability to incorporate biological matter (e.g. growth factors or proteins) or antibiotics in the coating during its processing.[43] Electrodeposition of HAp can be carried out in two different manners, depending on the building blocks used in the process – ions or nanoparticles. The latter has some potential benefits and has been successfully applied on a dental implant [48], but is beyond the scope of this work.

Eliaz et al. have carried out extensive research and development of CaP coatings on different substrates, including surface preparation of Ti and Ti6Al4V substrates to increase the adhesion of coatings [10–15, 19, 20, 27, 30, 44, 48]. In their first animal study [44], uncoated, PS-coated (Bio-Coat, Inc. Horsham, PA, USA), and ED’ed rods made of Ti6Al4V ELI were implanted into canine trabecular bone for 6 h, 7, and 14 days. While up to 7 days the PS’ed coatings showed higher bone apposition ratio (BAR), at 14 days after implantation the ED’ed coating showed similar BAR, much higher than that of uncoated Ti6Al4V. This kinetics was attributed to the higher crystallinity, and consequently – lower solubility, of the ED’ed coating. The finely textured microstructure of ED’ed coatings appeared to provide significant advantage for the integration of mineralized bone tissue into the coatings. However, since no surface preparation of the substrate rods was used, the adhesion of the ED’ed coating was poor. Following the development of a proper surface preparation procedure, grit-blasted and NaOH-treated uncoated Ti6Al4V rods, rods coated with EDHAp without alkali pretreatment, rods coated with EDHAp after alkali pretreatment, and rods coated with 80-μm-thick PSHAp (Eurocoating SpA, Mezzago, Italy) were press fitted into the intramedullary canal of mature New Zealand white rabbits and analyzed, both at the diaphyseal and at the metaphyseal zones, either 1 week or 12 weeks after surgery [43]. All rods were sterilized by gamma irradiation. NaOH-EDHAp exhibited a higher BAR value than the EDHAp at 1 week, and was as good as the commercial PSHAp at 12 weeks. The new bone area (NBA) value for NaOH-EDHAp at 12 weeks was the highest. The higher content of OCP in NaOH-EDHAp and the associated increase in the solubility of this coating in vivo were considered responsible for the enhanced osseointegration. In addition, the NaOH-EDHAp coating exhibited one third occurence of delamination compared to the commercial PSHAp coating.

To date no clinical investigation of NaOH-EDHAp-coated dental implants has been reported. The aim of the present clinical study was thus to evaluate the suitability and safety of this novel SBTC® surface treatment of dental implants, with a comparison to a commercially widely available electrochemical surface treatment that forms a mixture of brushite and hydroxyapatite (Bonit®).

## Materials and methods

### Trial design

The study followed a two-group, parallel design with a test group and a control group. A split-mouth design was applied, i.e., all participants received both types of implants. However, to offset potential operator-handedness effects, the enrolled participants were randomly assigned to two randomization groups (see below).

### Participants

All study procedures took place at Smile Dent Dental Center (Szeged, Csongrád-Csanád County, Hungary), an independent private office and registered clinical study site. Volunteers were sought through electronic and written media channels from all over Csongrád-Csanád County.

**Fig. 1:**
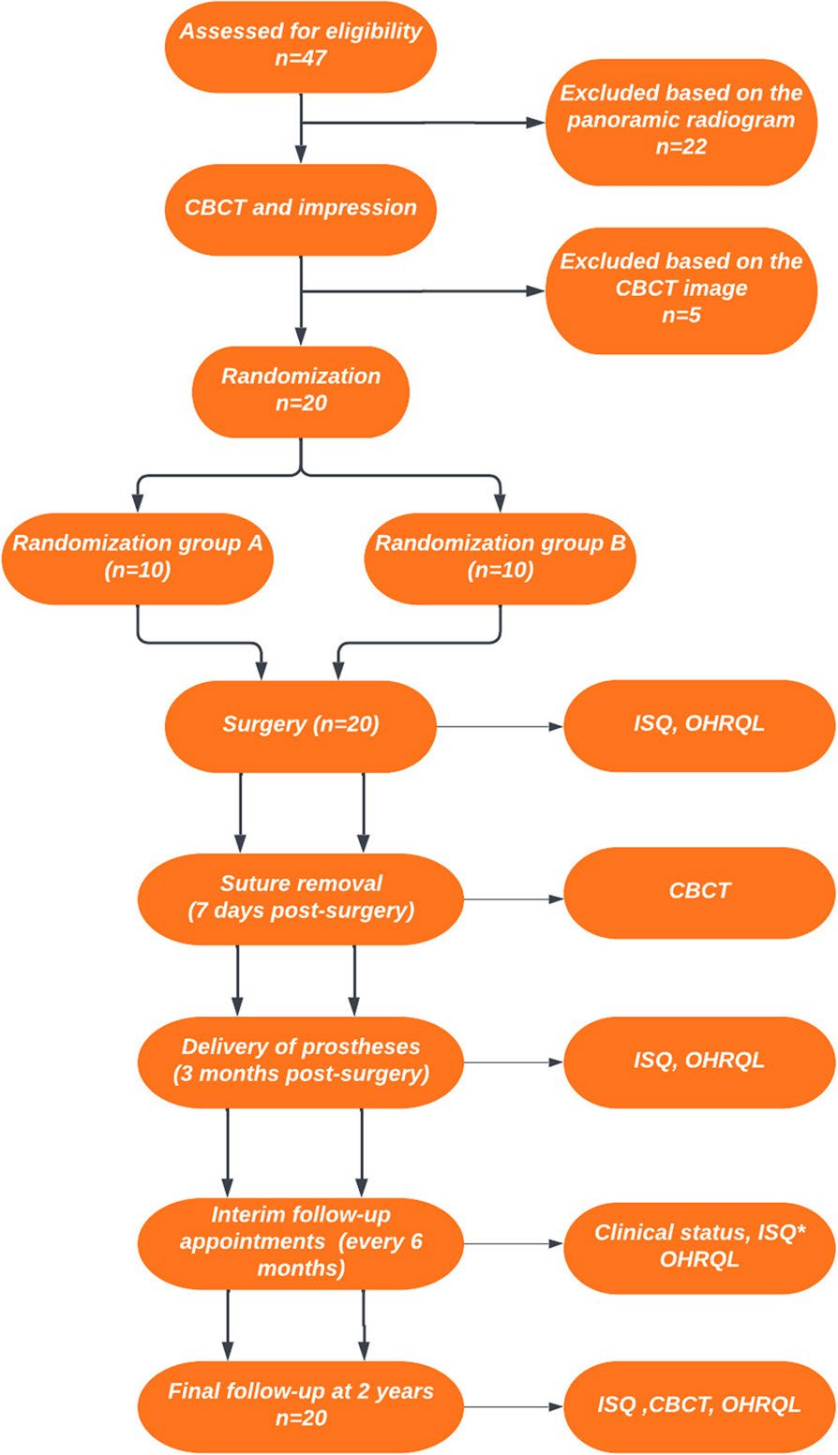
CONSORT flowchart. Asterisk (*) indicates that Osstell ISQ measurements were not performed in all visits in the interim visits period, only at 9 months after insertion, which falls into this period. OHRQL: Oral health-related quality of life (OHIP-14 questionnaire). The flowchart was generated using Lucidchart (Lucid Software, Inc., USA)

Recruitment of volunteers took place from June 13, 2019, through June 13, 2021. Follow-up ended on August 31, 2023. The flow of participants is shown in Fig. 1.

Twenty participants enrolled in the study, all of whom completed the study per protocol. Their data was subsequently subjected to analysis. Among these participants, 14 were females (70%), 6 were males (30%). The average age was 67.3 years, with a standard deviation of 7.6 years.

All patients underwent a standardized screening process conducted by the same examiner (R. K.). Initially, a panoramic radiograph was taken to assess bone dimensions and identify any signs of inflammation or anomalies. Patients with inadequate bone height or those requiring bone augmentation procedures were excluded from the study based on this panoramic assessment. Subsequently, a cone-beam computed tomography (CBCT) [49] scan was conducted to obtain precise bone measurements and assess bone quality. Patients with insufficient bone height or width, as well as those with non-healed extraction sockets or persistent inflammations, were excluded from the study. The inclusion and exclusion criteria were defined as follows:

Inclusion Criteria:

1. Complete edentulism in the mandible.
2. Assessment by the investigator that the patient is suitable for implantation based on adequate clinical conditions, including sufficient soft tissue and bone conditions, as well as appropriate occlusal position.
3. Clear consciousness of the patient, with an understanding of the planned intervention.
4. Effective communication between the patient and the examiner, with the ability to comprehend and comply with the requirements of the study protocol.
5. Provision of written informed consent.

Exclusion Criteria:

1. Pregnancy or lactation in women.
2. Women of childbearing potential, unless they use effective contraception methods until the completion of the final radiological examination and for an additional 4 weeks thereafter.
3. Presence of a disease (including but not limited to metabolic, hematological, renal, hepatic, pulmonary, neurological, endocrine, cardiac, and gastrointestinal diseases) that, in the investigator's opinion, significantly impacts the health of the individual being examined and/or poses an unacceptably high risk to the person undergoing implantation treatment.
4. A history of malignant disease within the 24 months preceding enrollment.
5. Known presence of HIV, hepatitis B, hepatitis C, or any other viral infection that, according to the Hungarian regulations, necessitate a higher level of protection and must be treated in a dedicated unit.
6. Medical or psychiatric conditions that, in the investigator's opinion, preclude the participant from adhering to the protocol or completing the study as per the protocol.
7. Participation in another interventional clinical study within the 6 months prior to treatment.
8. Known allergies to the implant, investigational template, or any of their components.
9. Limited mouth opening, as deemed by the investigator, making it impossible to complete the guided surgical procedure.
10. Increased pharyngeal reflex or reduced ability to tolerate intraoral manipulation.
11. History of radiotherapy or previous irradiation of the jawbones.
12. International Normalized Ratio (INR) > 2.5.
13. Immunocompromised status of the patient.
14. Previous or current bisphosphonate treatment.
15. Known history of alcohol or drug abuse.
16. Heavy smoking (≥ 20 cigarettes per day or equivalent forms of smoking).
17. Untreated periodontitis.
18. Local infection in the area planned for implant placement.
19. Inadequate dental hygiene, such as the presence of retained root fragments, plaque, calculus, or radiographically detectable potential periapical lesions (even in asymptomatic cases).
20. Insufficient or poor oral hygiene.
21. Transient infection (bacterial, viral, fungal or parasitic), with or without fever.
22. Patients with extraction at implant site up to 8 weeks prior inclusion

The study adhered to the principles outlined in the Helsinki Declaration of 1975, as amended in 2000, and followed the guidelines of Good Clinical Practice. Approval for the study protocol was obtained from the National Institute of Pharmacy and Nutrition of Hungary (Approval No. OGYÉI/36686/2019). The study is registered on clinicaltrials.gov under the identifier ID: NCT06034171, and the complete protocol can be obtained from the corresponding author upon request.

### Implant placement, dimensions, and implant surfaces

Regardless of surface treatment or randomization group, the implants were placed in the following positions: 32 to 35 (left side), 42 to 45 (right side). The following implant sizes were used: 3.75 × 11.5 mm (*n* = 13 in both groups) and 4.2 × 10 mm (*n* = 7 in both groups). The same participant always received the same implant size on both sides. All implants were of the P7D type from SGS Dental Holding (St. Gallen Switzerland), and were made of Ti6Al4V alloy. They were all grit-blasted with corundum (crystalline alumina), and just differed in their surface treatment – either SBTC® (test group, bath concentration × 39, SGS Dental, Budapest, Hungary) or Bonit® (control group, DOT Medical Implant Solutions GmbH, Rostock, Germany). The SBTC® process included acid treatment, soaking in NaOH, and electrodeposition for 30 min.

### Surface analysis

X-ray photoelectron spectroscopy (XPS) measurements were performed in UHV (2.5 × 10^–10^ Torr base pressure), using a 5600 Multi-Technique System (PHI, USA). The sample was irradiated with an Al-Kα monochromated source (1486.6 eV), and the outcome electrons were analyzed by a spherical capacitor analyzer, using a slit aperture of 0.8 mm in diameter. The sample was analyzed at the surface only, at 23º and 75º take-off angles. Charging was compensated with charge neutralizer. The binding energy (BE) of adventitious carbon at 285 eV was taken as an energy reference for all measured peaks. A low-resolution survey spectrum was taken over a wide energy range (0–1400 eV) to identify the elements present at the sample surface. High-resolution spectra were taken at pass energy of 11.75 eV at an increment of 0.05 eV/step. In order to identify unambiguously the specific CaP phases formed and determine their relative contents, the integrated intensity of the oxygen shake-up peaks was analyzed in combination with the Ca/P and O/Ca atomic ratios, following the procedure suggested by Eliaz et al. [50]. The calculation took into account that the coating was formed on a substrate with an oxygen-containing surface layer. Scanning electron microscopy (SEM) images were collected with a FEI Quanta 3D field emission scanning electron microscope (Hillsboro, Oregon, United States) operating at 20 kV 83.3 pA with 4 spot size and 20 kV 166 pA with 4.5 spot size.

### Interventions

#### Surgical planning and guide preparation

CBCT scans were acquired using and i-CAT® Next Generation scanner (Imaging Sciences International, Inc., Hatfield, PA, USA) and a standardized protocol, with the following settings: 120 kV, 5 mA, 9 s exposure time, voxel size: 250 µm, and a field of view (FOV) of 110 mm. A C-silicone impression (Zetaplus, Zhermack, Italy) was taken of the patient's lower arch in a plastic tray (Hi-Tray, Zhermack, Italy), which had been customized with individual guttapercha markers to enhance visibility in X-rays. Subsequently, two CBCT scans were acquired: one with the patient wearing the impression, and another solely of the impression itself. To ensure accurate positioning of the impression during exposure, the patient bit down on the tray. Both scans were then transmitted online to the service provider, where they were registered, and a case ready for planning was sent back to the surgeon. The surgeon planned the intervention via the surgical planning software (SMART Guide, dicomLAB Dental, Szeged, Hungary). The surgical templates were manufactured by 3D Printing, using a multijet technology printer (ProJet MD 3510, 3D Systems, SC, USA). Identical implant lengths and diameters were planned for both sides in all surgical procedures.

#### Pre-surgical procedures

All participants received education on the significance of maintaining proper oral hygiene and were provided with personalized guidance. The local prophylactic protocol was adhered to, involving the administration of either 2,000 mg of amoxicillin/clavulanic acid orally or 600 mg of clindamycin orally, 60 min prior to surgery. Following the application of local anesthetics, participants were instructed to perform a 30-s rinse with a 0.2% chlorhexidine solution (Corsodyl®, GlaxoSmithKline, Brentford, United Kingdom). Subsequently, the surgical procedures were carried out by the same experienced surgeon (M.Á.A.).

#### Surgical procedures

A crestal incision was created at the implant recipient site, followed by the elevation of a full-thickness flap. Upon access preparation, the surgical template was positioned, and all osteotomies were conducted using the template as guidance. The sole non-guided phase of the procedure was the implant insertion itself. The Universal Guided Kit (dicomLAB Dental, Szeged, Hungary) was utilized for performing the osteotomies. Implant placement adhered to the manufacturer's guidelines and employed the same physiodispenser used for osteotomy procedures (Implantmed Plus, W & H Ltd., Bürmoos, Austria). Subsequent to implant insertion and the initial stability assessment (as described below), a cover screw was introduced, and the flap was sutured-shut using non-absorbable silk monofilament sutures (Silkam®, BBBraun, Melsungen, Germany). Sutures were removed within 7 ± 3 days post-surgery.

#### Prosthetics and follow-up

All implants were exposed after a three-month healing period, and following a repeated implant stability quotient (ISQ) assessment [51], the patients received their full-arch dentures anchored by locators (SGS Dental Holding, St. Gallen Switzerland). Subsequently, the patients were monitored for a duration of two years following these procedures to evaluate the study’s outcomes.

### Outcomes

The primary outcome was implant stability (ISQ) over time. This was measured with an Osstell Beacon device (Osstell, Göteborg, Sweden). Measurements were performed in two directions in all cases, bucco-lingual (BL) and mesio-distal (MD), according to the manufacturer’s instructions. Measurements were taken at insertion, at loading (3 months), and then 9 months and 2 years post-insertion. As per the manufacturer's guidelines, an ISQ value exceeding 70 (on a scale of 0 to 100) indicates high stability, while an ISQ ranging from 60 to 69 indicates a medium level of stability, and an ISQ value falling below 60 is indicative of low stability [52].

Supplementary measurements were conducted by placing 10 SBTC®-treated and 10 implants with the control surface treatment into hard artificial bone using two different drilling protocols. The implants were inserted into polyurethane blocks with a density of 40 PCF (Nacional Ossos, Jaú, SP, Brazil). The block properties are defined by ASTM F1839-08.

The secondary outcome was the change in the level of the crestal bone around the implants, expressed in millimeters. A postoperative CBCT was taken with the same settings as described before, within 10 days after implant insertion, and at 2-year follow-up. Using the software of the CBCT system (Imaging Sciences International Inc., Hatfield, PA, USA), bone was measured in four sites (buccal, distal, lingual, and mesial), as per the method of Patil and Seow [53]. As bone level implants were applied, the insertion level was defined as the baseline (zero level) for the two-year measurements. Consequently, if during the two-year follow-up evaluation the implant's position was found to be lower than the initial bone level (resulting in negative values), it was considered indicative of bone gain. Conversely, if the implant's position was above the bone level (resulting in positive values), it signified bone loss.

As an ancillary measure unrelated to surface treatments, we evaluated the participants' oral health-related quality-of-life (OHRQL). This assessment involved the use of the standardized Hungarian version of the OHIP-14 questionnaire [54–56]. The questionnaire was administered before surgical intervention, 10 days after prosthesis delivery, and at 6 months, 12 months, 18 months, and 24 months after implant insertion. The OHIP-14 questionnaire covers 14 potential oral health issues across seven domains: functional limitations, physical discomfort, psychological distress, physical impairment, psychological impairment, social challenges, and handicaps. Each item is rated on a scale of 0 to 4, with 0 representing 'never' and 4 representing 'very often'. Higher scores indicate increased discomfort and lower quality-of-life in the specific area assessed. An individual's cumulative score ranges from 0 (complete comfort) to 56 (complete discomfort). To determine the OHRQL, we calculated the summed OHIP-14 score for each participant at each appointment and then determined the mean of these sums for each appointment. This approach allowed representing the average OHRQL of the entire study population at each visit.

### Sample size

Sample size estimation for the number of implants with the test and control surface treatments was conducted using G*Power 3.1.9.7 (Universität Düsseldorf, Germany) for analysis via repeated measures analysis of variance (ANOVA) within-factors. The specific parameters employed in this estimation included an effect size (*f*) of 0.22, a significance level (α) of 0.05, a power (1-β) of 0.95, one group being examined, four measurements taken per subject, a correlation coefficient among repeated measures (ρ) set at 0.5, and a non-sphericity correction factor (ε) of 1. Based on these parameters, the initial calculation indicated that a minimum sample size of 46 implants per surface treatment was necessary, which was subsequently rounded up to 50 implants per group to account for potential loss. However, during an interim analysis, it became evident that the initial estimation of effect size was underestimated. This underestimation could be attributed to the limited availability of studies employing similar designs and surface treatments for reference. Surprisingly, the observed effect size was larger than initially anticipated, resulting in the achievement of the desired statistical power for the repeated measures test with just 20 implants per group. Consequently, the study's final sample size was determined to be 20 implants per group.

### Randomization

The randomization to offset the potential effect of operator-handedness was performed using the block randomization module of Jamovi 2.3.21 (The Jamovi Group), with a block size of 2. This block randomization approach ensured that each block contained two implants – one SBTC® and one control. In randomization group A, participants received the test implant on the right side of their mandible and the control implant on the left side. In randomization group B, the opposite arrangement was employed. The randomization was carried out for 20 blocks, ensuring that all participants were assigned to a randomization group. Consequently, each randomization group included 10 participants, and the 40 implants (20 test and 20 control) were evenly distributed between the randomization groups.

The randomization sequence generated by the software was then printed, the blocks were labeled with A or B based on the implant arrangement (as described above), cut out, and placed into opaque envelopes. These envelopes were opened at the time of implant placement, and the randomization group was immediately recorded in the participant's documentation.

### Blinding and statistical analyses

In this study, blinding of the data analyst was implemented to ensure the objectivity and integrity of the data analysis process. The dataset provided to the data analyst was coded, with the study groups designated as "1" and "2," and the randomization groups labeled as "A" and "B". Consequently, the data analyst possessed knowledge solely of numerical identifiers, with no access to the actual study groups, preserving the concealment of treatment assignments. The data analyst received specific instructions regarding the analyses to be conducted. Subsequently, after the completion of the prescribed calculations, the blinding was lifted to enable the analyst to assist in the accurate interpretation of the data, should such assistance be required.

Statistical analyses were performed using Jamovi 2.3.21 (The Jamovi Group) and SPSS 26.0 (IBM, USA). Demographic data underwent descriptive analysis. Categorical variables were presented as counts and relative percentages, while continuous variables were summarized with counts, means, 95% confidence intervals, medians, minimums, and maximums. To compare parameters related to the implants between the two randomization groups, the Kruskal–Wallis H test was employed. The results indicated no statistically significant differences, confirming the successful randomization process in effectively mitigating any potential influence of the surgeon's skill. For the primary outcome variable (implant stability over time), the assumption of normality was not met for all variables according to the Shapiro–Wilk test. Consequently, Friedman ANOVA along with Durbin-Conover post-hoc pairwise comparisons was utilized for the repeated measures analysis. Effect sizes, calculated using Kendall’s W, were also reported. Regarding the secondary variable, bone level change over the entire 2-year observation period was assessed at four positions (as described above) for both study groups. As the assumption of normality was not met for all cases based on the Shapiro–Wilk test, the Kruskal–Wallis test was used to compare the two groups in terms of bone level change at these four positions. Lastly, the mean OHIP-14 scores across multiple appointments over time were compared using Friedman ANOVA with Durbin-Conover post-hoc pairwise comparisons. Statistical significance was defined as *p* < 0.05 for all analyses.

## Results

### Implant stability

Descriptive statistics are shown in Table 1 and Table 2. In the case of BL measurements [61], a significant change between T1 and T4 was found for both the control (χ^2^ = 50.1, mean change: 8.25) and the test surfaces (χ^2^ = 52.1, mean change: 13.3). In both cases: df = 3, *p* < 0.001, Kendall’s W = 0.835. The Durbin-Conover post-hoc analysis revealed that the change was significant between each time point. In the case of MD measurements [57], the same pattern was found. The change between T1 and T4 was significant for both the control (χ^2^ = 51.3, Kendall’s W = 0.855, mean change: 7.90) and the test surfaces (χ^2^ = 51.0, Kendall’s W = 0.850, mean change: 13.70). In both cases: df = 3, *p* < 0.001. The Durbin-Conover post-hoc analysis revealed that the change was significant between each time point.

**Table 1:** Descriptive statistics of the ISQ values measured in the *BL* direction across the four time points. T1: immediately after insertion; T2: at loading, 3 months after insertion; T3: 9 months after insertion; T4: 2 years after insertion; C: control group; T: test group

		**T1**	**T2**	**T3**	**T4**
Mean (95% CI)	**C**	77 (74.2–79.9)	80.8 (78.7–83.0)	83.1 (81.7–84.5)	85.3 (84.0–86.6)
	**T**	71.4 (68.1–74.7)	76.5 (72.9–80.1)	82.2 (80.7–83.7)	84.7 (83.2–86.1)
Median (min–max)	**C**	78.5 (60–85)	82 (67–87)	83 (78–89)	85.5 (78–90)
	**T**	71.5 (57–83)	79 (60–85)	82.5 (75–86)	85 (75–89)

**Table 2:** Descriptive statistics of the ISQ values measured in the *MD* direction across the four time points. T1: immediately after insertion; T2: at loading, 3 months after insertion; T3: 9 months after insertion; T4: 2 years after insertion; C: control group; T: test group

		**T1**	**T2**	**T3**	**T4**
Mean (95% CI)	**C**	77.5 (74.0–81.1)	81 (78.8–83.1)	83.2 (81.2–85.2)	85.5 (84.0–86.9)
	**T**	72.3 (68.1–76.4)	77.4 (73.8–81.0)	82.8 (81.4–84.1)	85.9 (84.3–87.5)
Median (min–max)	**C**	80 (60–85)	82 (68–87)	84 (70–89)	86 (74–89)
	**T**	75.5 (46–83)	79.5 (60–86)	83 (75–86)	86 (76–94)

**Fig. 2:**
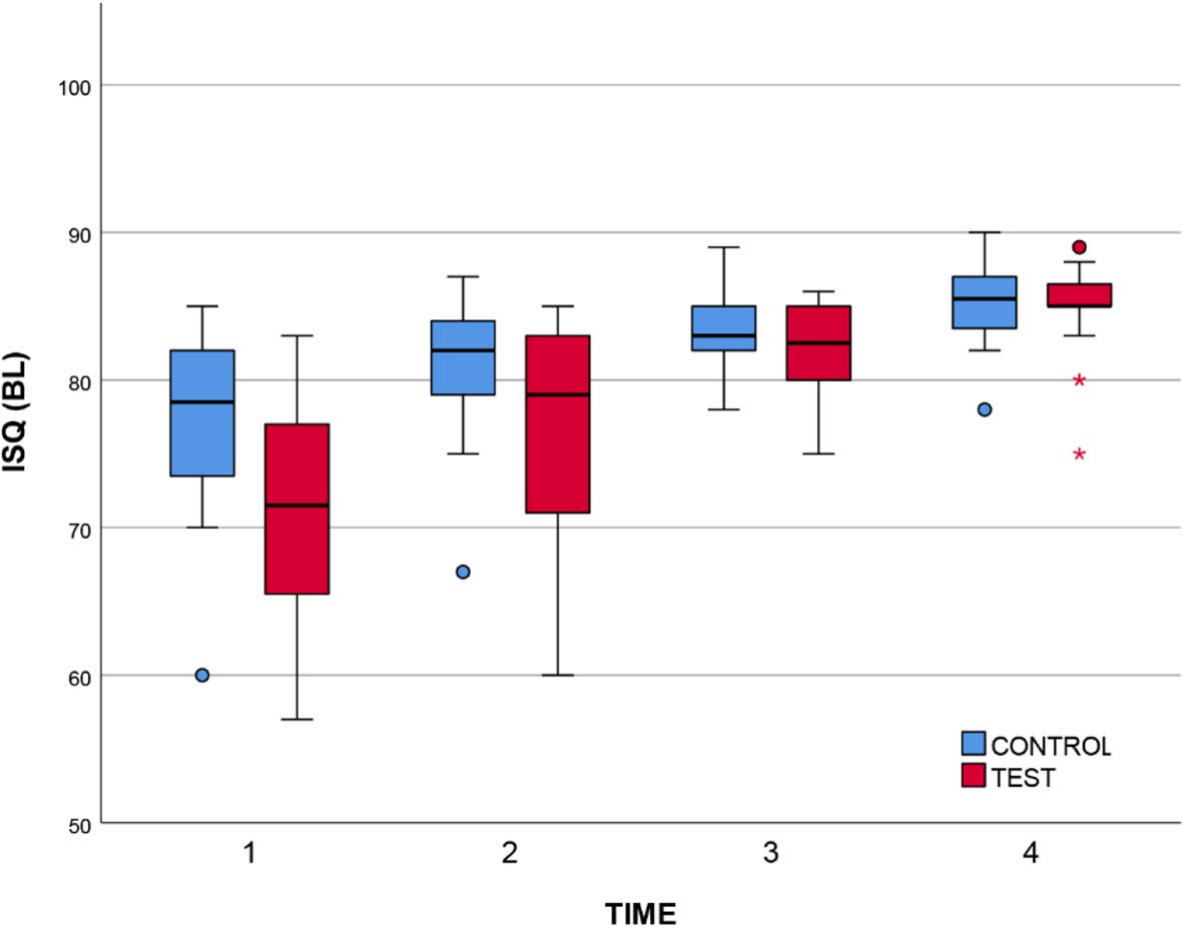
A boxplot summary of the ISQ results by study group (BL direction). Circles indicate outliers, stars indicate extreme outliers

**Fig. 3:**
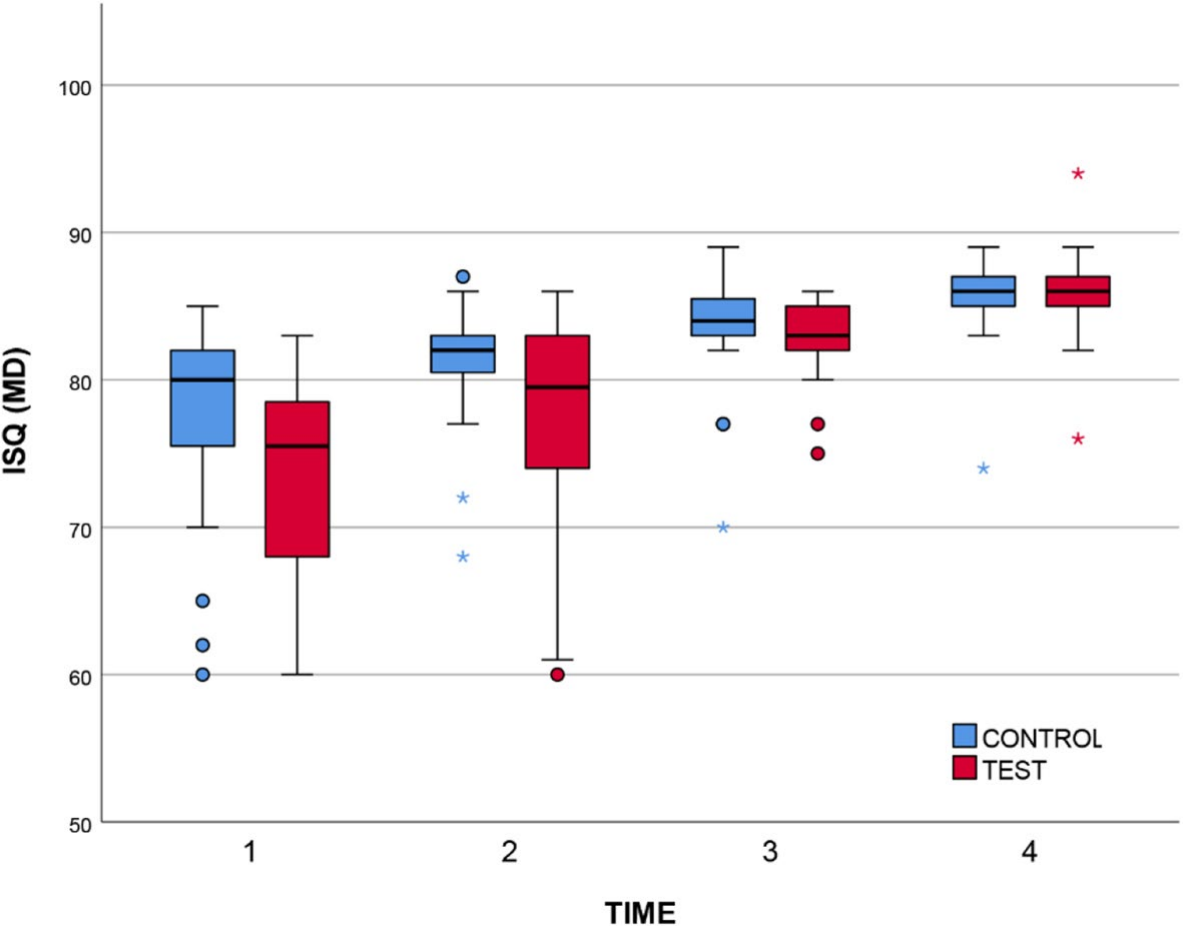
A boxplot summary of the ISQ results by study group (MD direction). Circles indicate outliers, stars indicate extreme outliers

Characteristically, both the BL and MD measurements indicated a slight advantage of the control surface at T1, which started to disappear by T2 and was eliminated by T4 (see Fig. 2 and Fig. 3). By the end of the 2-year observation period, the study groups were identical in terms of stability, both groups showing high stability. In all cases, irrespective of the study group, the implants exhibited sufficient stability to permit loading at T2. From Tables 1 & 2 it is evident that the ISQ values were within the range of 71.4 and 86 for T1–T4 in both directions. According to the scale provided in Sect. 2.6, values exceeding 70 indicate high stability.

### Bone level change

Bone level changes were individually calculated for each participant in each study group. The descriptive statistics illustrating bone level changes for each study group and measurement position are summarized in Table 3. As described in Sect. 2.6, negative values denote bone gain, while positive values denote bone loss. As the Shapiro–Wilk test indicated that the measurements recorded for the four positions did not follow a normal distribution, the two study groups were compared with the Kruskal–Wallis test. No statistically significant difference was found for any of the positions.

**Table 3:** Bone level change over the 2-year observation period (change in millimeters). C: control group, T: test group

		**Buccal**	**Distal**	**Lingual**	**Mesial**
Mean (95% CI)	**C**	0.81 (0.61–1.01)	0.79 (0.51–1.06)	0.49 (0.13–0.85)	0.65 (0.47–0.83
	**T**	0.57 (0.37–0.76)	0.52 (0.27–0.76)	0.46 (0.22–0.70)	0.36 (0.08–0.65)
Median (min–max)	**C**	0.76 (0–1.85)	0.67 (0.07–2.12)	0.60 (-1.83–1.54)	0.60 (0.18–1.53)
	**T**	0.57 (-0.3–1.37)	0.45 (-0.42–2.07)	0.30 (0–1.81)	0.49 (-1.77–0.9)

### Oral health-related quality of life (OHRQL)

The results of the OHRQL measurements are summarized in Table 4. It is evident that almost immediately after the delivery of the implant-retained prostheses, the patients experienced a significant improvement of OHRQL (*p* < 0.001, Friedman ANOVA with Durbin-Conover post-hoc pairwise comparisons), and the difference remained significant until the last appointment, with significant changes between the appointments. Neither adverse events nor.

**Table 4:** Mean OHIP-14 scores at each appointment when OHRQL was assessed. Only those appointments are shown when the questionnaire was administered, and the appointments are numbered accordingly. The OHIP-14 scores range from a minimum of 0 (absolute comfort with no discomfort in any addressed domain) to a maximum of 56 (absolute discomfort where all addressed domains cause the maximum degree of discomfort)

OHIP appointment	Mean ± SD	Time point
1	14.4 ± 13.2	Before implant surgery
2	4.8 ± 7.2	10 days after prosthesis delivery
3	3.20 ± 0.52	30 days after 2nd appointment
4	4.20 ± 0.52	6 months after implant surgery
5	5.15 ± 0.50	12 months after implant surgery
6	6.15 ± 0.50	18 months after implant surgery
7	7.15 ± 0.50	24 months after implant surgery

implant loss were reported during the 2-year follow-up period.

### The implants and their surface analysis

**Fig.4:**
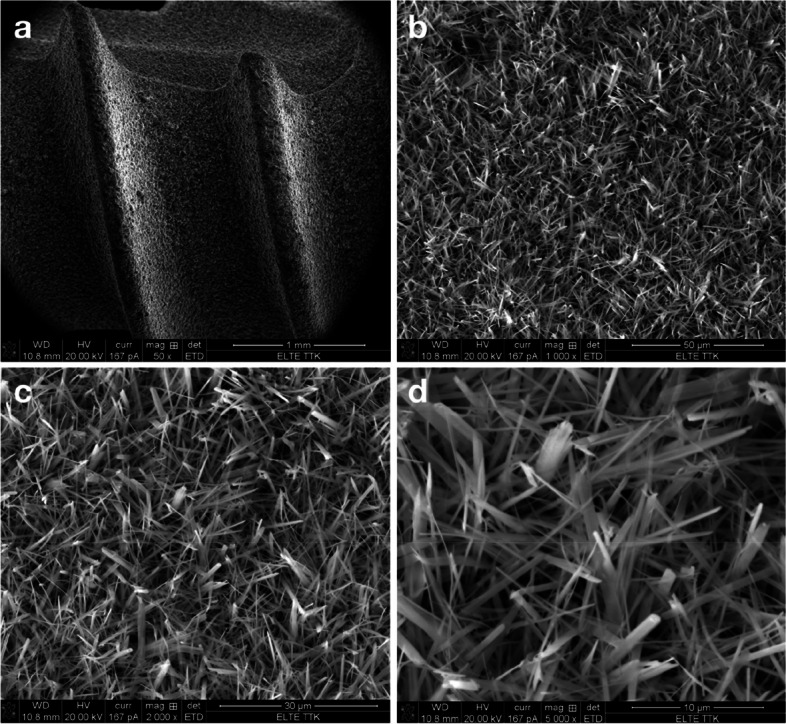
Scanning electron microscopic images of the test surface (SBTC) at 50x (**a**), 1000x (**b**), 2000x (**c**) and 5000x (**d**) magnification. Surface coverage between threads (**a**) and needle-like surface morphology (**b**-**d**) of the SBTC® coating. The scanned implant was from the same series as implanted in the participants

**Fig.5:**
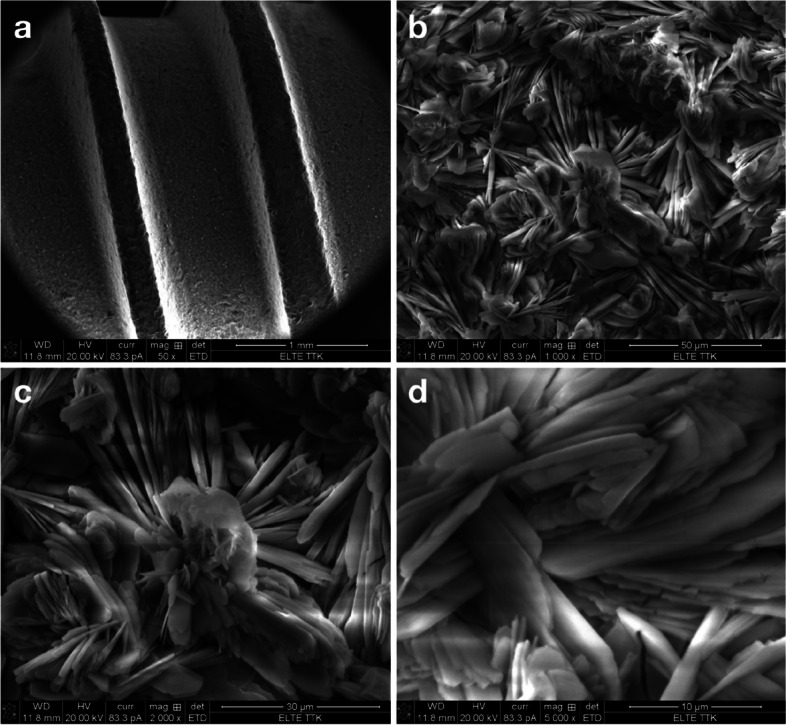
Scanning electron microscopic images of the control surface at 50x (**a**), 1000x (**b**), 2000x (**c**) and 5000x (**d**) magnification. Surface coverage between threads (**a**) and platelet-like surface morphology (**b**-**d**) of the reference (control) coating. The scanned implant was from the same series as implanted in the participants

Figure 4 shows SEM images of coated implants in the test group. Figure 4a reveals good coverage by the SBTC® coating around two threads of an implant. Figure 4b-d reveals the surface morphology of this coating. Such needle-like crystals are characteristic of nano β-tricalcium phosphate (TCP) [58], although it could be argued against this option that this coating was deposited from aqueous solution, and no complementary high-temperature heat treatment was conducted [10]. Different bath composition, ED bath configuration, voltage, and deposition time compared to previous work of Eliaz et al. may all result in the formation of different phases and different surface morphologies, e.g. no prismatic hexagonal bars [10, 20, 30] in the present work. Figure 5 shows SEM images of coated implants in the control group. Figure 5a reveals good coverage by the control coating around threads of an implant. Figure 5b-d reveals the surface morphology of this coating. Such platelets composed of whiskers are typical of brushite (DCPD) [59, 60]. Optical micrographs of the cross-section revealed that the control coating was non-uniform in thickness (12.6 ± 4.6 µm). Advanced XPS analysis revealed that the SBTC® coating consisted of 55% HAp and 45% OCP (atomic percentages). The presence of OCP enhances the osseointegration in vivo [10]. XPS analysis also showed that the control coating consisted of brushite and HAp. The brushite content varied between 60 and 53%, lower values prevailing deeper into the coating.

## Discussion

Based on our results, the SBTC®-treated implants demonstrated reliable and stable clinical performance, and no instances of implant loss or adverse events occurred during the 2-year follow-up period. Their performance and safety did not significantly differ from the control surface treatment, which is already used commercially.

The primary outcome of the study focused on implant stability over time, assessed through resonance-frequency measurements. In this regard, we observed that immediately after placement, the primary stability of SBTC® implants lagged slightly behind that of the control implants. However, the stability of SBTC® implants subsequently exhibited a steeper increase, ultimately reaching essentially the same level as that of the control implants by the end of the 2-year follow-up period. It is noteworthy that at 3 months post-implantation (at the time of loading), both types of implants displayed load-bearing capabilities, rendering the initial disparity practically insignificant within the framework of this protocol. Immediately after their insertion, SBTC® implants exhibited ISQ > 60, which is considered the normal value for the initial stability of implants used for clinical applications [61]. Nevertheless, we wanted to make sure that the differences in primary stability between the two groups did not stem from some intrinsic characteristic of the implants under examination. Thus, in a post-hoc analysis, we placed implants from both the test and the control groups (of the same batch as used in the patients) into artificial bone. Regardless of surface treatment or drilling protocol, high stability values (ISQ > 70) were obtained. This suggests that the ISQ values of the placed implants may be attributed to a particular characteristic of the study population rather than to an intrinsic characteristic of the surface treatment. Shim et al. [62] recently reported significantly higher variability in implant stability among elderly patients. This, combined with the distinctive features of the new surface treatment, could have contributed to the observed outcome.

The secondary outcome of interest in this study was the change in bone level. Pathological bone loss is typically defined when bone loss exceeds 2 mm [63–65]. Neither of the groups in our study exhibited such significant bone loss. Documented changes in bone levels can vary widely in the literature. Becker et al. [66] reported an annual bone loss rate of 0.1 mm, while Cakarer et al. [67] documented 0.4 mm of bone loss over 2 years, and de Carvalho et al. [68] observed 1 mm bone loss over 5 years. To narrow down this range somewhat, it is useful to compare our results to studies that used the same prosthetic superstructure as ours. Patil et al. [53] reported a bone loss of 0.46 ± 0.4 mm around locator-retained dentures in the first year, and 0.9 ± 0.8 mm with different implant sizes. The same authors [69] documented an average bone loss of 0.67 mm within one year in another locator study. Kutkut et al. [70] reported 0.65 ± 1.69 mm bone loss in the first year with immediate loading, but with a similar loading protocol to our study (loading after 3 months), they observed 1.33 ± 1.47 mm bone loss. Elsyad et al. [71] documented 0.98 mm bone loss in the case of locator-retained dentures one year after loading. Thus, our results are essentially in line with the literature. It is noteworthy, however, that in both test and control groups, instances of bone gain were observed. This phenomenon has previously been documented in some animal experiments, particularly with the use of the control surface treatment. Schwartz et al. [72] demonstrated that the resorbable CaP layer significantly enhances bone/implant contact. Schiegnitz et al. [37] found that CaP-coated surfaces on supracrestal inserted implants exhibit vertical osteoconductive characteristics and increase the bone/implant contact at the implant shoulder. Therefore, the observation of bone gain on the control surface was not entirely novel. It appears, however, that the SBTC® surface may also have a similar effect. As for the stability of this surface feature, it cannot be definitively determined based on the results of this study, as the protocol was not designed to assess this aspect, and the sample size was not calculated with this consideration. Nevertheless, it is advisable to monitor this aspect in future research endeavours.

The results derived from the ancillary OHIP-14 questionnaires provide unequivocal evidence of a notable enhancement in OHRQL as a result of prosthetic treatment. Importantly, this outcome is independent of the study groups, underlining the substantial amelioration in the quality of life experienced by participants due to prosthetic rehabilitation. It is noteworthy that throughout the 2-year follow-up period, while utilizing dentures, a mild decline in OHIP scores is discernible. However, this diminishment is likely unrelated to the implants themselves, but rather associated with the wear and tear of the locator dentures. Throughout the follow-up period, only essential prosthetic interventions were undertaken, including retentive component (locator nylon insert) replacement for two participants and re-lining in one. Despite the potential challenges inherent in comparing OHIP-14 results across diverse studies, our findings attest to excellent OHRQL. For instance, Brandt et al. [73] assessed OHRQL in connection with locator dentures and reported an OHIP-14 score of 10.4 ± 4.45.

While it can be considered a limitation that our follow-up period was limited to two years, it is noteworthy that according to the literature, the most critical period for bone loss occurs within the first year [74]. Therefore, our 2-year follow-up provides reliable data on the behavior of the surfaces, osseointegration, potential bone loss, and bone position. It can also be argued that another limitation of this study is that we did not assess the initial healing with the baseline control CBCT. However, the research aimed to compare the two surface treatments, and we did not intend to obtain absolute results regarding bone loss occurring in the period following initial bone remodeling.

## Conclusions

A randomized controlled clinical trial of dental implants was conducted to compare the clinical properties of the same type of implant with two different electrochemically deposited CaP coatings – SBTC® and a commercially available control surface treatment. It was concluded that the SBTC® surface treatment functions clinically with a similar level of reliability as the substantially equivalent control surface treatment. Patients' OHRQL significantly improved after denture delivery and remained stable throughout the follow-up. No complications or adverse events were observed.

## Data Availability

No datasets were generated or analysed during the current study.

## Abbreviations

ANOVA: Analysis of variance
BAR: Bone apposition ratio
BL: Bucco-lingual
CaP: Calcium phosphate
CBCT: Cone-beam computed tomography
CONSORT: Consolidated Standards of Reporting Trials
ED: Electrodeposition
FOV: Field of view
Hap: Hydroxyapatite
ISQ: Implant stability quotient
MD: Mesio-distal
OCP: Octacalcium phosphate
OHRQL: Oral health-related quality of life
PHAp: Precipitated hydroxyapatite
PS: Plasma spray
SAM: Self-assembled monolayer
SBTC: Smart Bioactive Trabecular Coating
SEM: Scanning electron microscopy
XPS: X-ray photoelectron spectroscopy
